# Self-Healable, Fast Responsive Poly(ω-Pentadecalactone) Thermogelling System for Effective Liver Cancer Therapy

**DOI:** 10.3389/fchem.2019.00683

**Published:** 2019-10-18

**Authors:** Huihui Shi, Hong Chi, Zheng Luo, Lu Jiang, Xian Jun Loh, Chaobin He, Zibiao Li

**Affiliations:** ^1^Department of Materials Science and Engineering, National University of Singapore, Singapore, Singapore; ^2^Shandong Provincial Key Laboratory of Molecular Engineering, School of Chemistry and Pharmaceutical Engineering, Qilu University of Technology (Shandong Academy of Sciences), Jinan, China; ^3^Fujian Provincial Key Laboratory of Innovative Drug Target Research and State Key Laboratory of Cellular Stress Biology, School of Pharmaceutical Sciences, Xiamen University, Xiamen, China; ^4^Institute of Materials Research and Engineering, A^*^STAR (Agency for Science, Technology and Research), Singapore, Singapore

**Keywords:** hydrogel, drug formulation, polymer synthesis, cancer therapy, self-healable, temperature responsive hydrogel, biodegradable (co)polymers

## Abstract

A polyurethane based thermogelling system comprising poly(ω-pentadecalactone) (PPDL), poly(ethylene glycol) (PEG), and poly(propylene glycol) (PPG), termed as PDEP, was synthesized. The incorporation of PPDL lowers critical micelle concentration (CMC) as well as critical gelation concentration (CGC) of the novel copolymers compared to commercial Pluronic^®^ F127. The thermogels showed excellent thermal stability at high temperature up to 80°C, fast response to temperature change in a time frame of less than second, as well as remarkable self-healing properties after being broken at high strain. *In vitro* drug release studies using docetaxel (DTX) and cell uptake studies using doxorubicin (DOX) show high potential of the hydrogel as drug reservoir for sustainable release profile of payloads, while the *in vivo* anti-tumor evaluation using mice model of hepatocellular carcinoma further demonstrated the significant inhibition on the growth of tumor. Together with its excellent biocompatibility in different organs, the novel PDPE thermogelling copolymers reported in this work could potentially be utilized as *in situ*-forming hydrogels for liver cancer therapy.

## Introduction

Thermoresponsive hydrogels, also known as thermogels, are an important class of physically crosslinked hydrogels whose aqueous polymer solution undergoes reversible sol-gel transition upon temperature change depending on the delicate balance between hydrophilicity and hydrophobicity (Liow et al., 2016). Especially, considerable attention has been drawn to thermogel systems with gelation temperature range of 10–40°C for biomedical applications such as minimally invasive drug delivery, injectable tissue engineering, wound healing, 3D cell culture and prevention of post-surgical adhesion (Moon et al., 2012).

Pluronic, a triblock copolymer of PEG and PPG, is a well-known thermogelling system which has been approved by Food and Drug Administration (FDA) for decades and attractive for *in situ* drug delivery and wound healing due to its excellent biocompatibility and tunable transition temperature (Wu et al., 2016a). However, Pluronic gels have been reported to demonstrate poor mechanical properties and be prone to erosion, usually persisting for <1 day *in vivo*. Meanwhile, they are not biodegradable and generally require high critical gelation concentration (CGC), which may result in side effects from accumulation (Yu et al., 2009). These disadvantages limited the potential application of Pluronic systems, and thus, much efforts have been devoted to the modification of Pluronic copolymers. Jeong et al. coupled Pluronic^®^ F127 with diphenylalanine which could form coordination bonds with metal ions Zn^2+^, and obtained thermogels with modulus increasing from 15–21 to 24–28 kPa and durability of gel against water-erosion prolonging from 24 to 60 h at 37°C (Kim et al., 2017). Park et al. modified triblock PEG-PPG-PEG copolymers with D-lactide or L-lactide oligomers on both sides. The hydrogels formed from the two-component copolymer solution not only exhibited sol-gel transition between 10 and 40°C, but much lower CGC value, greatly enhanced mechanical strength and improved stability in aqueous environment due to the stereocomplex formation between D-lactide and L-lactide oligomers (Chung et al., 2008). Besides, incorporation of another hydrophobic block via step growth polymerization to drive the self-assemble of amphiphilic block copolymers by hydrophobic interactions has been a popular strategy nowadays to give polyurethane based Pluronic derivates with enhanced mechanical properties and decreased CGC values. Biodegradable polyesters including poly(lactic acid) (PLA) (Loh et al., 2008; Wu et al., 2016c), polycaprolactone (PCL) (Li et al., 2012; Zheng et al., 2017; Liu et al., 2019), and polyhydroxyalkanoate (PHA) (Li Z. et al., 2008; Wu et al., 2016b; Wee et al., 2017; Zhu et al., 2018; Jiang et al., 2019) are mostly selected as the third segment to produce desired thermogelling copolymers, while some cases based on polycarbonates (Loh et al., 2012a; Chan et al., 2018) are also reported.

Cancer is a leading cause of death worldwide, among which liver cancer ranks as the sixth most common type of cancer and contributes to the second largest percentage of cancer mortality (McGlynn et al., 2015). Chemotherapy is one of the most important means in cancer treatment nowadays, killing cancer cells by using cytotoxic drugs such as docetaxel (DTX) and doxorubicin (DOX) (Norouzi et al., 2016; Li et al., 2017; Yang D. P. et al., 2017). A major drawback of traditional chemotherapy is the non-specificity, which often results in low drug efficacy and damages to normal cells and tissues. Alternatively, localized chemotherapy based on various drug delivery system such as hydrogels (Xing et al., 2016), nanoparticles (Sun et al., 2014), micelles (Amjad et al., 2017), and liposomes (Eloy et al., 2016) have been widely investigated in recent years. Thermogels are regarded as one of the most promising candidates as they can simply be administrated via subcutaneous injection and form gels *in situ* quickly at physiological temperature which could increase the solubility and stability of drugs *in vivo* and serve as a sustaining drug delivery depot to targeted tumor site (Liow et al., 2016).

In this work, we design a novel polyurethane based thermogelling copolymer by copolymerizing poly(ω-pentadecalactone) (PPDL), which has been reported to possess good biocompatibility as well as excellent mechanical properties (Xiao et al., 2018), with PEG and PPG. The molecular properties, micellar properties and gel properties of the synthesized copolymers were investigated. Furthermore, the potential of the developed thermogels as anti-tumor drug delivery carrier were further explored through a series of *in vitro* and *in vivo* biological experiments.

## Experimental Section

### Materials

Poly(ethylene glycol) (PEG, *M*_n_ = 2,000), poly(propylene glycol) (PPG, *M*_n_ = 2,050), dibutyltin dilaurate (DBT) (95%), 1,6-hexamethylene diisocyanate (HDI) (98%), ω-pentadecalactone (98%), ethylene glycol (99.8%), dibutyltin oxide (DBTO) (98%), 1,6-diphenyl-1,3,5-hexatriene (DPH), Pluronic^®^ F127 (PEG_100_PPG_70_PEG_100_ triblock polymer), phosphate buffered saline (PBS), docetaxel (DTX), and doxorubicin (DOX) were purchased from Sigma-Aldrich (Singapore). Organic solvents including anhydrous toluene, diethyl ether, isopropanol (IPA), tetrahydrofuran (THF) and ethanol were of ACS grade and obtained from commercial sources. Dulbecco's modified Eagle's medium (DMEM), penicillin, streptomycin sulfate and fetal bovine serum (FBS) were purchased from Life Technology Co., Ltd. (Waltham, MA, USA). Thiazolyl blue tetrazolium bromide (MTT), hematoxylin and eosin staining kit were purchased from Yeasen Biotechnology Co., Ltd. (Shanghai, China). Dialysis tubing (MWCO 3,500 *D*a) was purchased from Spectrum Laboratories (USA). PEG, PPG, ω-pentadecalactone and ethylene glycol were dried under vacuum overnight before use. PBS buffer (0.01 M, pH = 7.4) were prepared by dissolving PBS powder in deionized water. Other materials were used as received.

### Synthesis of Poly(ω-Pentadecalactone) Diol (PPDL-Diol)

PPDL-diol was synthesized by the ring opening polymerization of ω-pentadecalactone (Kratz et al., 2009). Twenty gram ω-pentadecalactone (83.2 mmol) was heated to 130°C and then 0.17 g ethylene glycol (2.7 mmol) and 0.07 g DBTO (0.28 mmol) were added under argon atmosphere as initiator and catalyst, respectively. The reaction mixture was dissolved in THF after stirring for 21 days and precipitated in a 5-fold excess of an ethanol/water mixture (50/50 vol%). The resultant PPDL diol was washed with ethanol and vacuum dried at room temperature with a yield of 85% and an average molecular weight of *M*_n_ = 6,310 g·mol^−1^.

### Synthesis of Poly(PPDL/PEG/PPG Urethane) (PDEP) Copolymers

A series of poly(PPDL/PEG/PPG urethane)s were synthesized through a process similar to Chan's study (Chan et al., 2018). The molar ratio of segments PEG and PPG was fixed at 2:1 and the feed weight percentage of PPDL content was set at 2, 5, and 8 wt%, respectively. The resultant copolymers were denoted as *n*PDEP copolymers, where *n* represents for the feed weight percentage of PPDL component, PD for PPDL, E for PEG, and P for PPG. Typically, 10 g starting materials in total, including 6.53 g of PEG (*M*_n_ = 2,000, 3.3 mmol), 3.27 g of PPG (*M*_n_ = 2,050, 1.6 mmol), and 0.2 g of PPDL-diol (*M*_n_ = 6,310, 3.2 × 10^−5^ mol) were charged into a 250 mL round bottom flask. Dissolve reactants with 100 mL of anhydrous toluene and remove most of the solvent by rotary evaporation with about 10 mL of toluene left. The mixture was stirred and heated up to 110°C under argon atmosphere and then 1.25 mL of HDI (7.8 mmol) and two drops of DBT was injected into the flask as chain extender and catalyst, respectively. The mixture turned viscous gradually and extra 20 mL of anhydrous toluene was added each time when it was hard for the magneton to rotate. After 24 h reaction, products were precipitated from diethyl ether, re-dissolving in IPA and followed by dialysis in deionized water for 72 h. The final pure 2PDEP was obtained by freeze dry. 5PDEP and 8PDEP were prepared through this method, too. Copolymer yields were 70–75%.

### Molecular Characterization

^1^H nuclear magnetic resonance (NMR) and ^13^C NMR spectra were conducted on JEOL 500 MHz NMR spectrometer (Tokyo, Japan) at room temperature. Deuterated chloroform (CDCl_3_) was used as solvent for all the samples and chemical shifts were referenced to the solvent peaks at 7.3 and 77 ppm, respectively. Fourier transform infrared (FT-IR) spectra of the copolymer films dissolved in the chloroform coated on KBr tablets were conducted on Spectrum 2000 Perkin Elmer FT-IR spectrophotometer at room temperature. FT-IR spectra were obtained by signal averaging 32 scans at resolution of 4 cm^−1^.

### Thermal Analysis

Thermogravimetric analysis (TGA) was performed on TA Instruments TGA Q500 analyzer (USA) with a heating rate of 20°C·min^−1^ from room temperature to 800°C under a dynamic nitrogen stream (flow rate = 60 mL·min^−1^). Differential scanning calorimetry (DSC) thermal analysis was performed on photo differential scanning calorimeter (PDSC, Q100, TA Instruments, USA) and indium was used for calibration. The sample was equilibrated at −80°C for 5 min and heated up to 200°C at the rate of 20°C·min^−1^, then equilibrated at 200°C for 2 min and cooled down to −80°C at the rate of −20°C·min^−1^. Measurement was conducted twice and data from the second run were used for analysis in case of thermal history in the first run.

### Critical Micelle Concentration (CMC) Determination

Aqueous copolymer solution (10 mg·mL^−1^) was prepared and gradient diluted to obtain samples with a series of concentration. Twenty microliter DPH methanol solution (0.6 mmol·L^−1^) was added into every 1 mL aqueous copolymer solution and incubated equilibrated at 4°C overnight. UV-vis spectra of the copolymer/DPH solution in the range of 320–460 nm were measured by UV-Vis spectrophotometer (UV-2501 PC, Shimadzu, Japan) at 25°C. Difference in absorbance at 378 and 400 nm (A_378_-A_400_) vs. the logarithmic concentration was plotted to determine the CMC value.

### Particle Size Analysis

Dynamic light scattering (DLS) measurements were conducted on Zetasizer Nano ZS (Malvern Instruments, Southborough, MA) at 633 nm laser light and 173° scattering angle. Particle size and size distribution were characterized by intensity. Aqueous copolymer solutions (1 mg·mL^−1^) were passed through a 0.45 μm pore-sized syringe filter before measurements. Reversibility of micelle was evaluated by reversible transition test at 25 and 70°C for 5 cycles, with 15 min equilibration time between each measurement run.

### Sol-gel Transition Phase Diagram Determination

Two milliliter aqueous copolymer solution of a given concentration ranging from 6 to 20 wt% were prepared in 4 mL vials and placed at 4°C for 24 h to achieve full dissolution. The samples were equilibrated in water bath with designated temperature for 5 min ranging from 4 to 80°C at interval of 2°C. Critical gelation temperature were defined by the formation of firm gels which kept intact when inverted the vials for a while.

### Rheological Studies

The rheological measurements of the thermogels were conducted on TA Instruments Discovery DHR-3 hybrid rheometer (New Castle, DE, USA) fitted with a flat-plate geometry (SST ST 40 mm diameter) and a temperature-controlled peltier base plate. Storage modulus (G') and loss modulus (G”) were measured under different types of oscillatory tests. Amplitude sweeps (strain of 0.01–100% and frequency of 1 Hz) and frequency sweeps (frequency of 0.1–100 Hz and strain of 1%) were both performed at 37°C. Temperature ramps were performed between 25 and 37°C and temperature sweeps were performed from 4 to 80°C at a heating rate of 5°C·min^−1^, both with strain fixed at 1% and frequency fixed at 1 Hz. Self-healing properties of the thermogels were evaluated by amplitude sweep test at two predetermined strain for 10 cycles, 300 s at low strain and 120 s at high strain, with temperature fixed at 37°C and frequency fixed at 1 Hz.

### *In vitro* DTX Release From PDEP Thermogel

One milligram docetaxel was dissolved in an acetone solution together with 10 mg polymer material, dispersed in a phosphate buffer solution, and self-assembled in water to form micelles. The acetone was removed by dialysis, and the micelle solution was added to a polymer-containing PBS solution (2 mL) under low temperature conditions, stirred at a low temperature until thoroughly mixed, and then gelatinized at 37°C. Transfer it to a 15 ml tube, add 10 ml of PBS solution pre-warmed to 37^o^C, place in a shaker, release the drug at 100 rpm, collect 500 μl of solution per day, and re-add the same volume of fresh PBS solution. The collected solution was detected by high performance liquid chromatography (HPLC) with a mobile phase of 50% acetonitrile and a detection wavelength of 227 nm.

### Cytotoxicity Analysis

Hepatoma cells HepG2 cells (American type culture collection, ATCC) were cultured in high glucose medium containing double antibody and 10% fetal bovine serum at 37°C, 5% CO_2_ (MacDiarmid et al., 2009). Cytotoxicity analysis was performed using the classical MTT method. HepG2 cells in good growth state were seeded in 96-well plates at a density of 5,000 cells per well, and cultured at 37°C, 5% CO_2_ for 24 h. The cells were treated with different samples (PDEP group with the concentration of PDEP from 0 to 1,000 μg·mL^−1^, DTX group with the concentration of DTX from 0 to 50 μg·mL^−1^ and DTX/PDEP groups with the concentration of DTX from 0 to 50 μg·mL^−1^ and the concentration of PDEP from 0 to 250 μg·mL^−1^), and after 24 h, the configured MTT solution was added and incubated for 4 h. The results were detected by a microplate reader.

### Cell Uptake Analysis

HepG2 cells were placed in a 24-well plate containing glass slides at a density of 20,000 cells per well, and cultured at 37°C, 5% CO_2_ for 24 h, and the prepared sample solution (DOX group with the concentration of DOX at 1 μg·mL^−1^ and DOX/PDEP groups with the concentration of DOX at 1 μg·mL^−1^ and the concentration of PDEP at 5 μg·mL^−1^) was added for 2, 6, and 12 h, respectively. After that, the samples were washed away with PBS and fixed with 4% paraformaldehyde for 15 min, then mounted with a DAPI containing sealer and photographed with a confocal microscope Zeiss LSM5.

### *In vivo* Antitumor Effect

All animal experiments were carried out in accordance with the Animal Care Guidelines of Xiamen University under Protocol Number: XMULAC20190033. HepG2 cells in good condition were inoculated to the dorsal side of Balb/c nude mice at a density of 4 million cells per tumor. After the tumor has grown to the appropriate size, the mice are treated with PBS, 2PDEP (12 wt%), 5PDEP (14 wt%), 8PDEP (20 wt%), DTX (1 mg·ml^−1^), DTX (1 mg·ml^−1^)/2PDEP (12 wt%), DTX (1 mg·ml^−1^)/5PDEP (14 wt%), and DTX (1 mg·ml^−1^)/8PDEP (20 wt%) with three nude mice randomly divided into each group. The PBS group and DTX group were given twice a drug (5 mg·kg^−1^) every 2 weeks, and the hydrogel groups were given drug (5 mg·kg^−1^) once a week. The size of the tumor was recorded with a vernier caliper every other day, and the body weight was weighed. The tumor volume was calculated according to the formula of 1/2 × length × width^2^. After 2 weeks, the mice were sacrificed and the relevant tumor tissues were collected for the next step analysis.

### H&E Staining Analysis

The collected tissues and organs were subjected to gradient dehydration for 24 and 12 h with high glucose solutions of 15 and 30%, respectively, and frozen sections were cut at a thickness of 6 μm, followed by staining with hematoxylin and eosin staining for observation and analysis.

### Statistical Analysis

All charts and data processing were processed using origin 8 analysis software, the experimental data were expressed as mean and variance, and the significance analysis was analyzed using GraphPad 5.0.

## Results and Discussion

### Synthesis and Characterization of PDEP Copolymers

**Scheme 1:**
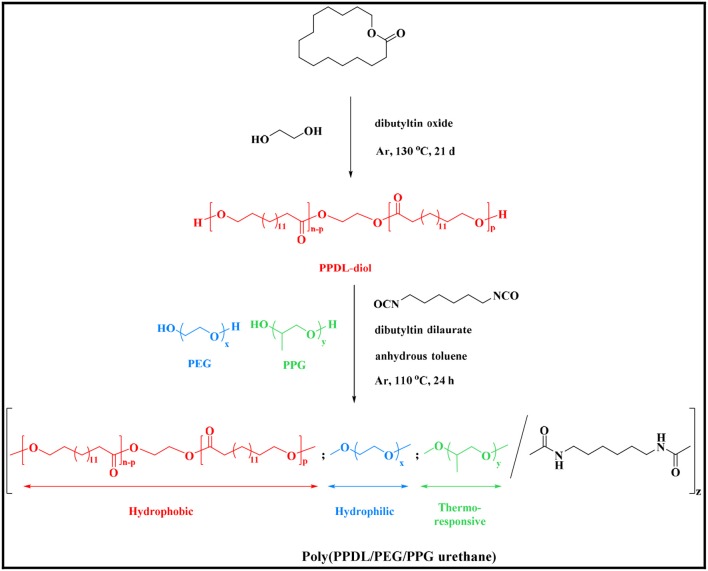
Synthetic routes of PDEP copolymers.

As shown in Scheme 1, PPDL-diol was first prepared via ring opening polymerization of ω-pentadecalactone at the presence of initiator ethylene glycol and catalyst DBTO. Then a series of random multiblock PDEP copolymers with different amounts of PPDL incorporated were synthesized via co-condensation of the macrodiols of PPDL, PEG, and PPG using an aliphatic diisocyanate HDI as coupling reagent in the presence of catalyst DBT linker.

**Figure 1:**
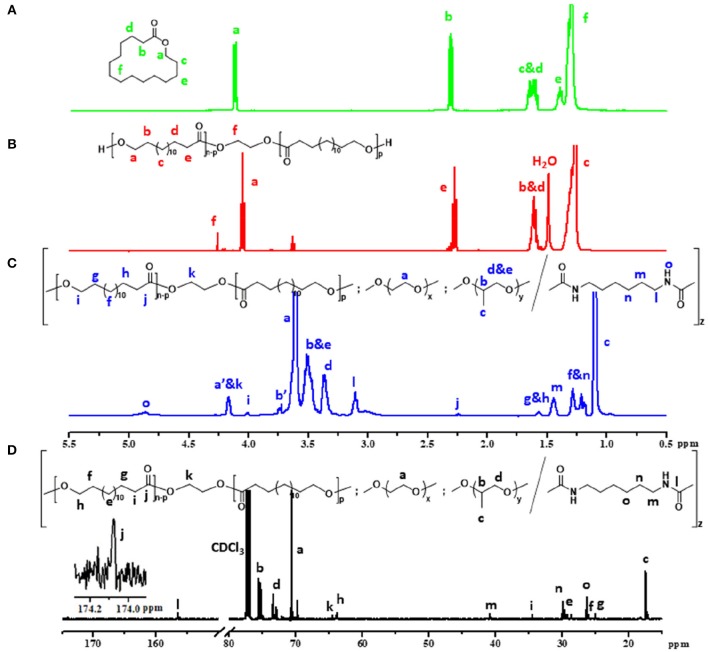
Five hundred megahertz ^1^H NMR spectrum of **(A)** ω-pentadecalactone, **(B)** PPDL-diol, **(C)** 2PDEP (a', b' represent protons on terminal unit of PEG and PPG segments which reacted with HMDI) and **(D)** ^13^C NMR spectrum of 2PDEP in CDCl_3_.

The chemical structure of the ω-pentadecalactone, PPDL-diol and PDEP copolymers were verified by ^1^H NMR and ^13^C NMR spectroscopy. According to Figures 1A,B, the specific peaks of PPDL-diol are almost consistent with its monomers except the signals at 4.3 ppm that belong to the methylene of ethylene glycol. And by comparing their integration values, the polymerization degree is estimated to be 26 and the average molecular weight of PPDL-diol is ~6,310 g·mol^−1^.

Figure 1C and Figure S1 show the typical ^1^H NMR spectrum of PDEP in CDCl_3_ with all proton signals belonging to PEG, PPG, and PPDL segments confirmed (Jiang, 2011; Li et al., 2012). In details, the signals corresponding to methyl protons of PPG are presented at 1.1 ppm while the signals corresponding to protons attached to backbone carbons in PPG are observed at 3.4 and 3.5 ppm. The signals at 3.6 ppm are assigned to methylene protons in repeated unites of PEG segments while the signals at 1.2 ppm are attributed to methylene protons in repeated unites of PPDL segments. The compositions of each component in the PDEP copolymers could be calculated from the integration ratio of distinguishable proton signals at 1.2, 3.4, and 3.6 ppm, and the results are shown in Table 1.

**Table 1:** Molecular characteristics and properties of PDEP copolymers.

**Copolymer**	**Feed ratio/g**			**Composition in copolymer/wt%**^**a**^			**Copolymer characteristics**	**Thermal properties**			
	**PPDL**	**PEG**	**PPG**	**PPDL**	**PEG**	**PPG**	**CMC^**b**^ (g·mL^**−1**^)**	**T_**d**_/°C^**c**^**	**T_**c**_/°C^**d**^**	**T_**m**_/°C^**d**^**	**T_**g**_/°C^**d**^**
2PDEP	0.2	6.53	3.27	2.9	68.3	28.8	7.24 ×10^−4^	335.48	30.57/−2.63	86.83/41.43	−57.12
5PDEP	0.5	6.33	3.17	5.0	67.1	27.9	7.85 ×10^−4^	313.98	30.12/−0.83	86.27/37.71	−58.26
8PDEP	0.8	6.13	3.07	9.5	63.8	26.7	7.10 ×10^−4^	304.59	31.04/2.46	86.34/36.82	−58.58

a *Calculated from ^1^H NMR spectroscopy results*.

b *Determined by the dye solubilization technique at 25°C*.

c *Obtained from TGA analysis*.

d *Obtained from DSC analysis*.

^13^C spectrum of 2PDEP is shown in Figure 1D. The signals at 17.3, 73.4, and 75.4 ppm are ascribed to methyl, methylene and methine carbon of PPG segments, respectively, and the signals at 70.6 ppm are attributed to methylene carbon of the PEG segments (Li et al., 2012). The signals at 174.0 and 34.5 ppm are attributed to the carbonyl carbon and methylene carbon alpha to the carbonyl group of PPDL segments, respectively, while the signals corresponding to the rest methylene carbon could be found between 25 and 30 ppm (Jiang, 2011). Meanwhile, the spectrum also presents signals generated from the HDI junction unit at 26.4, 30.0, 41.0, and 156.5 ppm, indicating that the polycondensation reaction was successful (Li et al., 2012).

**Figure 2:**
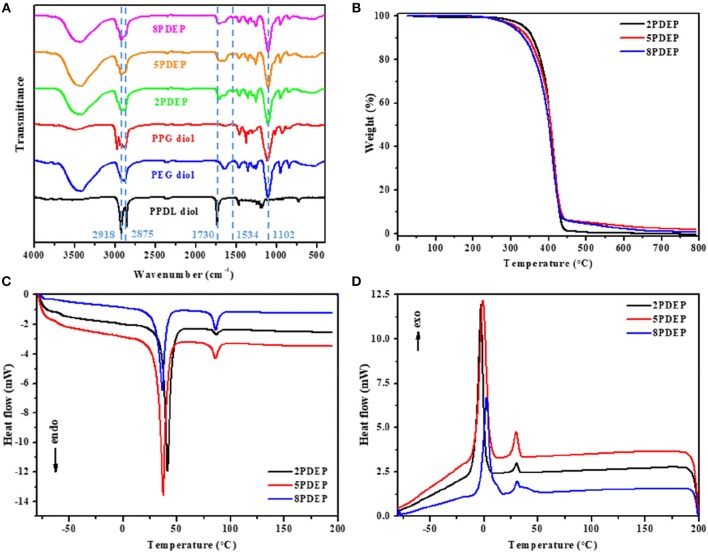
**(A)** FT-IR spectra of PDEP copolymers and macrodiols, **(B)** TGA curves of PDEP copolymers, **(C)** DSC heating curves of PDEP copolymers and **(D)** DSC cooling curves of PDEP copolymers.

FT-IR spectra of a series of PDEP copolymers and macrodiols of PEG, PPG and PPDL further confirm the successful synthesis of copolymers (Figure 2A). The FT-IR spectrum of PPDL-diol is typical of the stretching vibration of C=O in ester group whose absorption band is strong and sharp at 1,730 cm^−1^ (Pilate et al., 2018). Both PEG and PPG precursors present an intensive absorption band at 1,102 cm^−1^ due to the stretching vibration of C-O-C in the repeated unites (Loh et al., 2007). Absorption band for stretching vibration of saturated C-H in three macrodiols are exhibited at 2,875 and 2,918 cm^−1^. All these characteristic absorption bands are clearly observed in the FT-IR spectra of PDEP copolymers, confirming the presence of PPDL, PEG, and PPG segments. Additionally, the characteristic absorption band of HDI between 2,260 cm^−1^ and 2,280 cm^−1^ attributed to the stretching vibration of NCO disappears while a new small absorption band corresponding to the deformation vibration of N-H appears at 1,534 cm^−1^ in the FT-IR spectra of copolymers (Pilate et al., 2018), which gives evidence of the successful reaction between hydroxy groups in polymer precursors and isocyanate groups in HDI.

The thermal analysis of PDEP copolymers are conducted as well to understand the influence of the incorporation of PPDL and confirm the suitability of temperature for biomedical applications. The TGA curves shown in Figure 2B represent similar profile with F127 (Qin et al., 2013), but better thermal stability measured by decomposition temperature. Compared to commercial Pluronic^®^ F127 which starts to lose weight at around 220°C, all the synthesized copolymers are thermally stable below 300°C. Defined as the onset temperature at 5% weight loss, the decomposition temperature (T_d_) for 2PDEP, 5PDEP, and 8PDEP is 335.48, 313.98, and 304.59°C, respectively. The incorporation of the urethane linkages and the hydrophobic PPDL segments enhances the rigidity of the backbone as well as the intermolecular forces, contributing to the improvement in thermal performance for the copolymers together (Wang et al., 2015). But meanwhile, the addition of PPDL might compromise the regularity of the polymer chains to some extent, which counteracts the positive effects and makes the T_d_ of the copolymers decline with the increasing amount of PPDL (Yang S. et al., 2017).

As for the DSC results of PDEP copolymers, two melting peaks in heating curves (Figure 2C) and two crystallization peaks in cooling curves (Figure 2D) are observed for all copolymers, which might be attributed to the presence of two different types of crystalline domains rich in PPDL and PEG segments, respectively (Araneda et al., 2012). Meanwhile, the copolymers exhibit a single glass transition in heating curves, suggesting that the PPDL segments are likely to be thermodynamically miscible with PEG and PPG segments (Yeo et al., 2018). The crystalline temperature (T_c_), melting temperature (T_m_) and glass transition temperature (T_g_) of the copolymers are tabulated in Table 1. Compared with the value of T_c_ and T_m_ for PEG (35 and 53°C) and PPDL (85 and 90°C) in reference to literature (Martino et al., 2012; Kuru and Aksoy, 2014), T_c_ and T_m_ of the copolymers are both lowered as copolymerization depresses the regularity of polymer chains and reduces the crystallinity (Li B. et al., 2008). However, the influence of PPDL content on T_c_, T_m_, and T_g_ are not evident, probably because the PPDL content is too low and close to make a difference.

### Micellar Properties of Aqueous Copolymer Solutions

**Figure 3:**
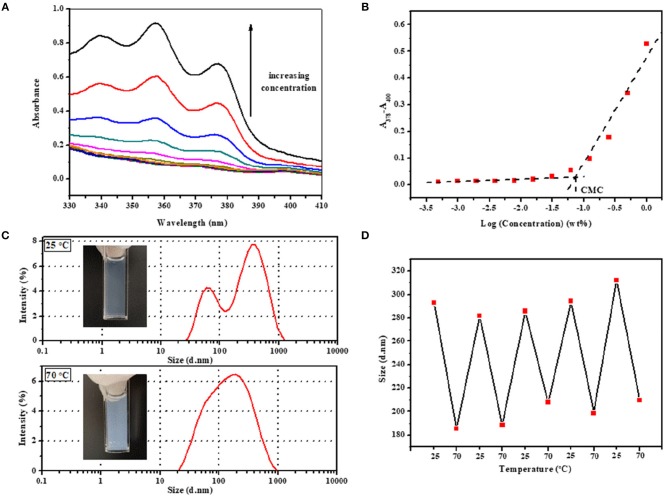
**(A)** UV–vis spectra of aqueous copolymer solution with concentration ranging from 0.005 to 10 mg·mL^−1^ at 25°C and **(B)** CMC determination for 2PDEP. **(C)** Real images and hydrodynamic diameter distribution by intensity of micelles at 25 and 70°C and **(D)** reversible transition in mean hydrodynamic diameter distribution of micelles between 25 and 70°C for 2PDEP aqueous solution with concentration of 1 mg·mL^−1^.

The PDEP copolymers are amphiphilic and able to form micelles in aqueous solution above the critical micelle concentration (CMC). The CMC values for these copolymers were determined by dye solubilization method at 25°C. The absorption coefficient of hydrophobic dye DPH in a hydrophobic environment is higher than that in a hydrophilic environment. When micelles are formed with increasing copolymer concentration, DPH molecules are incline to entering the hydrophobic core of the micelles and thus the absorbances of the aqueous copolymer solutions at 344, 358, and 378 nm increased (Figure 3A, Figures S2A,C) (Alexandridis et al., 1994). The point where the absorbance values display a sharp increase is defined as the CMC at which micelle formation occurs. As shown in Figure 3B and Figures S2B,D, the difference of absorbance at 378 nm and 400 nm (A_378_-A_400_) is plotted vs. the logarithmic concentration of the copolymers to determine the CMC values, which are tabulated in Table 1. In view that the CMC value for commercial Pluronic^®^ F127 with similar mass fraction of PEG and PPG is reported to be 2.5 × 10^−3^ g·mL^−1^ at 25°C by literature (Perry et al., 2011), the incorporation of hydrophobic PPDL segments makes a remarkable decrease of the CMC values for PDEP copolymers to around 7 × 10^−4^ g·mL^−1^ as a result of the enhanced hydrophobic interaction as well as driving force for self-assembly to achieve a state of minimum free energy. Nonetheless, the CMC values for the copolymers show no significant change with the increasing of the PPDL content, which might be ascribed to the relatively close PPDL content and the wide molecular distribution.

The micelles formed from PDEP copolymers are typically composed of a hydrophobic core and hydrophilic corona (Nakashima and Bahadur, 2006). The presence of PPG segments, well-known for exhibiting hydrophilicity at lower temperature and hydrophobicity at higher temperature, endows the copolymer micelles with thermal sensitivity (Shinohara et al., 2014). To investigate this property, the hydrodynamic diameter distribution by intensity for 2PDEP micelles in aqueous solutions (1 mg·mL^−1^) were investigated by DLS at two different temperatures (25 and 70°C). As shown in Figure 3C, at lower temperature, there are double peaks with partially overlap, one peak value of which is at around 60 nm and the other is at around 340 nm. The corresponding mean diameter and PDI of the micelles are 293.1 nm and 0.512, respectively. According to literature, hydrodynamic micelle diameter for commercial Pluronic^®^ F127 at 25°C has been observed as 30 nm or so with a single peak in size distribution (Desai et al., 2001). Thus, this result indicates that the incorporation of hydrophobic PPDL segments might lead to an increase in micelle size as well as aggregation of micelles to provide a favorable hydrophilic and hydrophobic balance. At higher temperature, the size distribution curve tended to be unimodal with the peak value at around 190 nm. The corresponding mean diameter and PDI of the micelles are 185.6 and 0.306, respectively. With elevated temperature, the PPG chains become more hydrophobic and tend to pack more tightly into the micelle core, resulting in smaller micelles, but meanwhile, the percentage of micellar aggregates increase since the PPG units are more liable to dehydrate and collapse with each other, supported by the significantly decreases in the optical transmittance of the copolymer solution from 25 to 70°C. The integration of these two effect makes the double peaks come closer and brings out one merged peak with an overall decrease in mean diameter by intensity eventually.

The reversibility of the hydrodynamic micelle size change triggered by temperature was characterized by DLS too. The transition of 2PDEP aqueous solution were conducted between 25 and 70°C for five cycles with 15 min for equilibrium before measurement each time. As shown in Figure 3D, the mean hydrodynamic diameter by intensity of the micelles decreases from 293 ± 10 nm at 25°C to much smaller value of 198 ± 10 nm at 70°C, exhibiting good reversibility upon temperature change due to the reversible hydrophilicity and hydrophobicity transition of PPG segments.

### Thermo-Responsive Sol-gel Transition and Gel Properties

**Figure 4:**
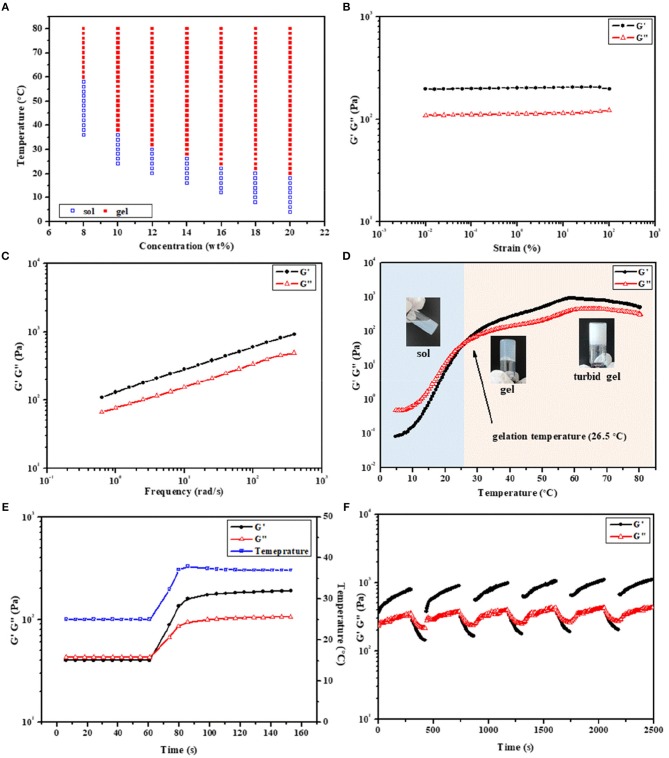
**(A)** Phase diagrams of 2PDEP in aqueous solutions. Storage modulus (G') and loss modulus (G”) of **(B)** amplitude sweep (0.01–100%, 1 Hz, 37°C), **(C)** frequency sweep (1%, 0.1–100 Hz, 37°C), **(D)** temperature sweep (1%, 1 Hz, 4–80°C) and **(E)** temperature ramp (1%, 1 Hz, 25 to 37°C) for 2PDEP aqueous solution (12 wt%) obtained from dynamic rheological analysis. **(F)** Self-healing cycle amplitude sweep (0.01 and 50%, 1 Hz, 37°C) for 8PDEP aqueous solution (16 wt%) obtained from dynamic rheological analysis.

Similar to thermogelling systems we have reported previously (Loh et al., 2012b; Wu et al., 2016c; Wee et al., 2017), PDEP copolymers in aqueous solutions render an increasing tendency to successively form micelles, micellar aggregates, and gels with temperature and copolymer concentration going up as a result of enhancing intra- and inter-micellar interactions. To investigate the gel formation ability of PDEP copolymers in aqueous, the tube inverting method was employed to determine their phase diagrams which underwent a monotonic increase of temperature from 4 to 80°C at interval of 2°C. Instead of typical sol-gel-sol transition with increasing temperature for many reported thermogelling systems resulted from the collapse of hydrogel networks, PDEP thermogels exhibit good stability at high temperature and demonstrated sol-gel-turbid gel transition (Figure 4A), which might be attributed to the strong association between hydrophobic PPDL and PPG segments and is supported by the thermal analysis results (Chan et al., 2018). Reverse phase transition were observed when the samples were cooled down from 80 to 4°C as well. Moreover, CGC values for 2PDEP, 5PDEP, and 8PDEP are around 7, 9, and 13.5 wt%, respectively, all lower than the CGC value for commercial Pluronic^®^ F127 which is around 17 wt% on account that the incorporation of hydrophobic PPDL provides greater driving force for the copolymers to self-assemble into gels at certain concentrations (Figure S3). However, it also appears that the raise of PPDL content in the copolymers causes the CGC values to increase. The reason is probably because that the increasing amount of PPDL gives rise to high viscosity of the copolymer solutions and impede segmental motions in the process of self-assembly, and thus increase inhomogeneity and defects in the networks, which are not firm enough to be regarded as gels for low concentration groups (Barshtein et al., 1995).

The rheological behaviors of PDEP thermogels were measured to investigate their thermo-responsiveness. Amplitude sweep between the strain range of 0.01–100% was conducted at 37°C first to determine the linear viscoelastic regime. The storage modulus (G') and loss modulus (G”) for 2PDEP aqueous solution with the concentration at 12 wt% are almost constant at all test strain, in which G' is much greater than G” and suggests the copolymer solution behaves a solid-like property at 37°C (Figure 4B). When the applied strain are higher above 100%, both G' and G” decline rapidly and a reversal of their relative position is observed at strain of around 180%, indicating the deformation of the thermogels (Figure S6A). Similar changes of G' and G” with increasing strain were observed for 5PDEP and 8PDEP (Figures S4A, S5A). Frequency sweep was also conducted between the frequency range of 0.1–100 Hz at 37°C and the results show positive dependence of G' and G” on oscillation frequency, where the copolymer solution is at gel state all the time and become stronger and stronger (Figure 4C, Figures S4B, S5B). This tendency could be explained by the time-temperature superposition principle for the viscoelastic behavior of polymers. Given that the intramolecular associations in thermogels would be strengthened and raise the value of both G' and G” at high temperature, which are supported by the result of temperature sweep (Figure 4D, Figures S4C, S5C), the increase of frequency is equivalent to the increase of temperature within certain range and therefore brings out the concomitant rise of G' and G”. The thermogelling transition of the copolymer solutions was verified by consecutive temperature sweeps between 4 and 80°C. With temperature varying from low to high, the G' and G” of the copolymer solutions keep increasing, resulting in a transition from liquid-like property to solid-like property. Gelation temperature for 2PDEP copolymers at the concentration 12 wt% is determined by the crossover of G' and G” curves. As the copolymer solution at this point actually presents a semi-solid state which is not yet firm, it is justified for the gelation temperature obtained from rheological studies (26.5°C) to be a bit lower than the value obtained from tube inverting method (32°C). Besides, it's noteworthy that PDEP thermogels demonstrate fast sol-gel transition in a time frame of less than second upon the change of temperature (Figure 4E, Figures S4D, S5D). As shown in Figure 4E, 2PDEP aqueous solution performs as liquid-like state (G' < G”) at 25°C and immediately converts to stable solid-like state (G'>G”) as the temperature was raised to 37°C, the fast responsiveness of which makes it advantageous in biomedical application such as minimally invasive *in situ* delivery system.

The self-healing properties of the thermogels were also evaluated by dynamic rheological analysis. Though the non-covalent interactions in the thermogels are strong enough for the formation of a gel, they were actually weak for the network when the gel is exposed to external force which is possible to break then. Thus, the ability of the materials to self-heal to original gel structure by the same physical interactions when the external stress is removed is practical in applications (Karim and Loh, 2015). As shown in Figure 4F, the thermogels formed from 8PDEP present rapid sol-gel transition and regular changing profile of G' and G” with the strain alternately varying between 0.01 and 50%, and are able to recover original strength gradually after being damaged by high strain. Similar phenomena are observed in the thermogels of 2PDEP and 5PDEP (Figures S6B,C), offering them prolonged lifetime as biomaterials *in vivo*.

### DTX Release From PDEP Thermogels *in vitro*

**Figure 5:**
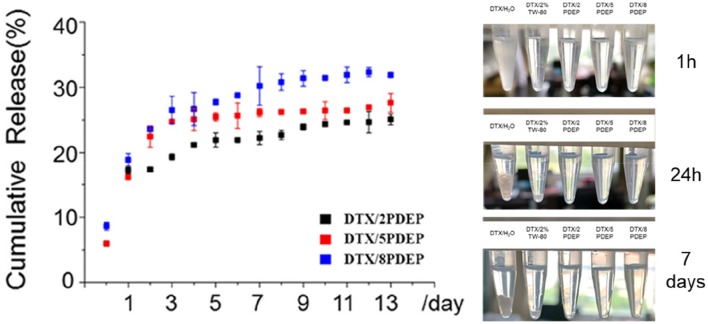
*In vitro* drug release profile of DTX released from PDEP micelle-hydrogel system.

Some chemotherapeutic drugs with good anticancer effect are often limited in clinical use due to their poor water-solubility. Improving the water solubility and stability of some hydrophobic drugs is of great significance for improving their anti-tumor effects. As is apparent from Figure 5, PDEP-wrapped DTX can significantly increase the water solubility of the hydrophobic DTX drug. Compared with the commonly used surfactant Tween-80, DPEP loaded DTX showed more stability, and no obvious DTX precipitation was observed after 1 week at room temperature

In order to verify whether the micelle-hydrogel system loaded with DTX drugs could continuously and effectively release the drug, we conducted a 14 day *in vitro* release experiment, and the experimental results are shown in Figure 5. There is a burst release of DTX occurred in the first 5 days, and then the drug release from the three hydrogels tends to be stable. As can be seen from the figure, after 14 days of drug release, the maximum drug release of 2PDEP, 5PDEP, 8PDEP thermogels was 25, 27, and 32%, respectively. All three hydrogels were able to release the drug continuously and effectively, and the drug release profile was similar.

### Cytotoxicity Analysis

**Figure 6:**
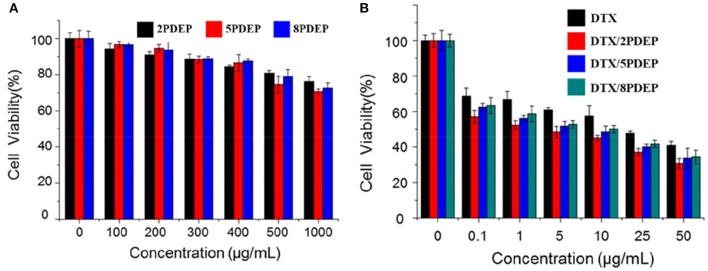
Cytotoxicity of different drug forms for HepG2 cells. **(A)** Different PDEP micelle forms; **(B)** different DTX drug forms.

The killing ability of PDEP micelles loaded with DTX drugs for liver cancer HepG2 cells was verified by using the classical MTT method. From Figure 6A, it could be seen that when the concentration of the PDEP copolymers reaches 1 mg·mL^−1^, the cell survival rate still has nearly 80% of cell survival. This shows that our material itself is not significantly toxic to cells. From Figure 6B, we can see that compared with individual DTX group, the killing ability of the drug against HepG2 cells in the DTX/PDEP group could be significantly improved. It might be because the formation of DTX/PDEP micelles could increase the solubility and stability of the drug and the amount of drug entering the tumor cell. Therefore, when the drug-loaded micelles are encapsulated into the PDEP thermogels, continuous drug delivery in the form of micelles and increasing amount of drug taken by tumor cells could be achieved, resulting in better anti-tumor effects.

### Cell Uptake Analysis

**Figure 7:**
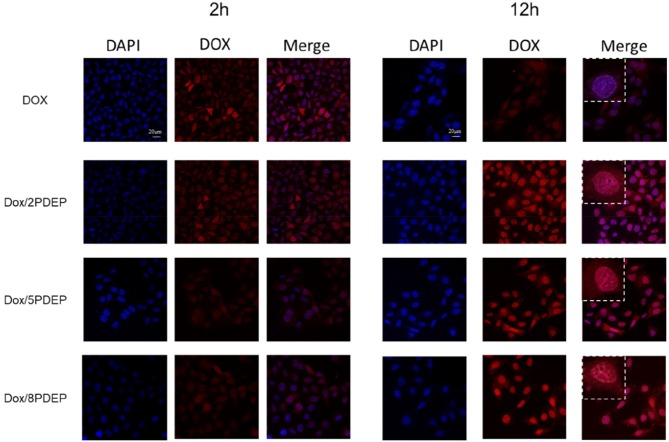
Confocal images of HepG2 cells incubated with DOX (1 μg·mL^−1^) and PDEP micelles (5 μg·mL^−1^) loaded with DOX (1 μg·mL^−1^) after 2 or 12 h.

To explore the ability of HepG2 cells to take in micelles, fluorescent DOX instead of DTX was used. Figure 7 shows the confocal images of hepatoma cell HepG2 incubated with DOX/PDEP micelles. Red fluorescence represents the drug DOX and blue fluorescence represents the DAPI stained nuclei. It could be seen that at 2 h, the HepG2 cells of individual DOX group shows obvious red fluorescence while the red fluorescence in the DOX/PDEP group was dim. It might be because that individual drug enters the cell mainly through diffusion while the drug encapsulated in copolymer micelles enters the cell mainly through endocytosis. The rate of the former manner is faster and brings about stronger red fluorescence in early period. At 12 h, the intensity of red fluorescence for DOX/PDEP group is significantly stronger than that of the individual DOX group, but there is no significant difference among three groups of PDEP. This might be due to the fact that the amounts of DOX that is swallowed into the cells are more than the drugs that are diffused alone, and the material might be able to increase the stability of the drug within the cell. From the confocal images, we could see that the cells are able to ingest the material well, and the material could increase the amount and stability of the drug into the cells, which is potential to be a good drug delivery carrier.

### Antitumor Effect *in vivo*

**Figure 8:**
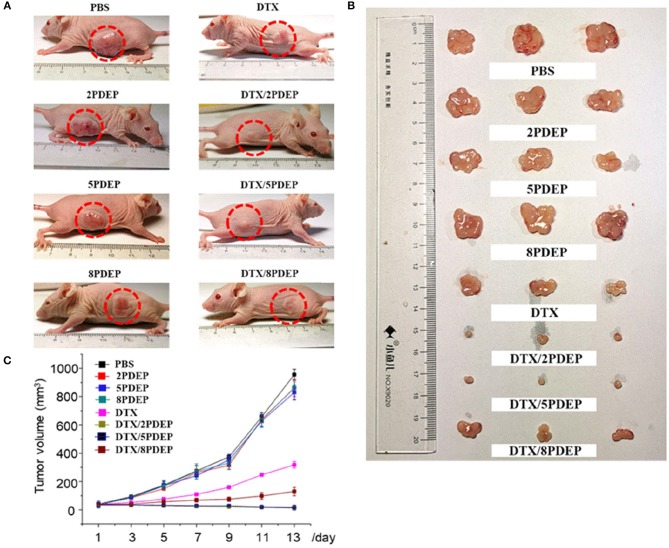
The therapeutic effect of the DTX-loaded micelle-PDEP thermogel system on xenograft tumor mice established with HepG2 cells. **(A)** Tumor in the body of mice. **(B)** Tumor isolated from mouse. **(C)** Tumor volume size curve.

To explore the *in vivo* anti-tumor effects of DTX-loaded micelle encapsulated in PDEP thermogels, we established a subcutaneous HepG2 liver cancer xenograft model for exploration. In initial stage, intratumoral injection of DTX/PDEP thermogels were implemented. When the tumor size on the back of the mouse reached 40 mm^3^, we started subcutaneous administration. The therapeutic effect after 14 day treatment is shown in Figure 8. It could be seen that the size of the tumor in PDEP alone group is close to that of controlled PBS group, and the final tumor size is about 900 mm^3^, indicating that a single material does not have the effect of inhibiting the tumor. Compared to the DTX alone group whose final tumor size is about 300 mm^3^, the DTX/PDEP group is able to significantly inhibit tumor growth. Among three copolymers, 2PDEP and 5PDEP work best and there is no significant difference between each them, both the final tumor size of which is about 15 mm^3^. The final tumor size of the DTX/8PDEP group is about 120 mm^3^, whose inhibition effect is relatively poor in comparison with other two copolymers. It might be because the rate of drug release in 8PDEP thermogels faster and imbalanced with the retention ability of drug in tissue, resulting in lesser release of the drug in later stage and thus poorer inhibition effect. As 2PDEP and 5PDEP thermogel systems could effectively achieve sustained drug release and effectively inhibit the development of HepG2 tumors, they are considered to have a good prospect in clinical application.

### H&E Staining Analysis

**Figure 9:**
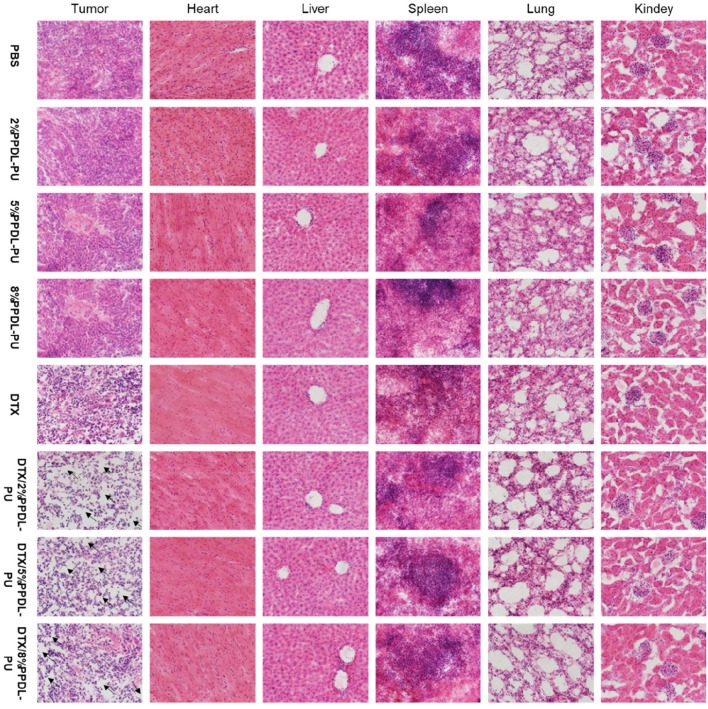
HandE staining analysis of the effects of different drug forms on tumors and major organs (heart, liver, spleen, lung, and kidney).

To further explore the effects of different drug forms on tumor tissues and other tissues and organs, we analyzed sections of tumor tissues and major organs by H&E staining. From the results shown in Figure 9, we could see that the tumor tissue sections of the PDEP alone group did not differ significantly compared with the PBS controlled group, further verifying that the individual materials could not kill the tumor cells. In the drug-administered group, it is obvious that there is a large area of tumor cell apoptosis in the tumor tissue section, and the number of tumor cells was significantly reduced, especially for DTX/2PDEP and DTX/5PDEP group. As for other organs (heart, liver, spleen, lung, and kidney) sections, all the groups have no significant difference compared with the PBS controlled group, further indicating that the hydrogel has a good biosafety.

## Conclusion

In this work, a series of multiblock poly(PPDL/PEG/PPG) urethane polymers were synthesized with good thermal stability and miscibility. The dilute PDEP copolymer aqueous solutions self-assembled into micelles with lower CMC values (~7 × 10^−4^ g·mL^−1^) compared to commercial Pluronic^®^ F127, which shrank and aggregated at elevated temperatures and exhibited good reversibility as characterized by DLS. In certain concentration and temperature, the PDEP copolymer aqueous solutions could form thermogels and kept stable even at temperature as high as 80°C. The CGC values of PDEP copolymers were related to the composition of PPDL segment and all lower than that of commercial Pluronic^®^ F127, among which 2PDEP copolymer gave the best gelation performance with CGC at 7 wt%. According to rheological results, the PDEP based thermogels presented fast response to temperature change and good self-healing properties after being broken by high strain. As for biocompatibility, individual PDEP copolymers displayed low toxicity both *in vitro* and *in vivo*. *In vitro* drug release studies showed continuous release of DTX from PDEP based thermogels for about 5 days with the cumulative amount up to 32%, and cell uptake studies demonstrated that the DOX loaded PDEP based micelles could increase the amount and stability of the drug entering the cells by endocytosis. Through *in vivo* anti-tumor effect studies, the growth of xenograft HepG2 tumor on mice was proved to be significantly inhibited by DTX loaded PDEP thermogel system, especially for 2DPEP and 5PDEP, while no damage were caused to other normal tissues. As all these results shown, the PDEP copolymers are promising to be a good drug delivery depot for chemotherapeutic applications.

## Data Availability Statement

The datasets generated for this study are available on request to the corresponding author.

## Ethics Statement

All animal experiments were carried out in accordance with the Animal Care Guidelines of Xiamen University under Protocol Number: XMULAC20190033.

## Author Contributions

HS: gel synthesis and manuscript writing. HC: materials characterization and discussion. ZLu: mice model built and bio-experiments. LJ: rheology evaluation. XL: technical advisor for drug formulation and discussion. CH: rational design of material composition and structure-property relationship. ZLi: technical advisor to overview the project, manuscript planning, and revision.

### Conflict of Interest

The authors declare that the research was conducted in the absence of any commercial or financial relationships that could be construed as a potential conflict of interest.
