# Supplementary material for: Self-Healable, Fast Responsive Poly(ω-Pentadecalactone) Thermogelling System for Effective Liver Cancer Therapy
Source: Front Chem. 2019 Oct 18;7:683. doi: 10.3389/fchem.2019.00683 (PMC6813430; doi:10.3389/fchem.2019.00683)
Supplement: Supplementary file 1 [file Table_1.DOCX]

**Supplementary Information**

**Self-healable, Fast Responsive poly(ω-pentadecalactone) Thermogelling System for Effective Liver Cancer Therapy**

Huihui Shi^a,#^, Hong Chi^b,#^, Zheng Luo^c^, Lu Jiang^d^, Xian Jun Loh^d^, Chaobin He^a,*^, Zibiao Li^d,*^

^a^ Department of Materials Science and Engineering, National University of Singapore, 9 Engineering Drive 1, Singapore 117576

^b^ Shandong Provincial Key Laboratory of Molecular Engineering, School of Chemistry and Pharmaceutical Engineering, Qilu University of Technology (Shandong Academy of Sciences), Jinan 250353, China

^c^ Fujian Provincial Key Laboratory of Innovative Drug Target Research & State Key Laboratory of Cellular Stress Biology, School of Pharmaceutical Sciences, Xiamen University, Xiamen 361102, China

^d^ Institute of Materials Research and Engineering, A*STAR (Agency for Science, Technology and Research), 2 Fusionopolis Way, Innovis, #08-03, Singapore 138634, Singapore


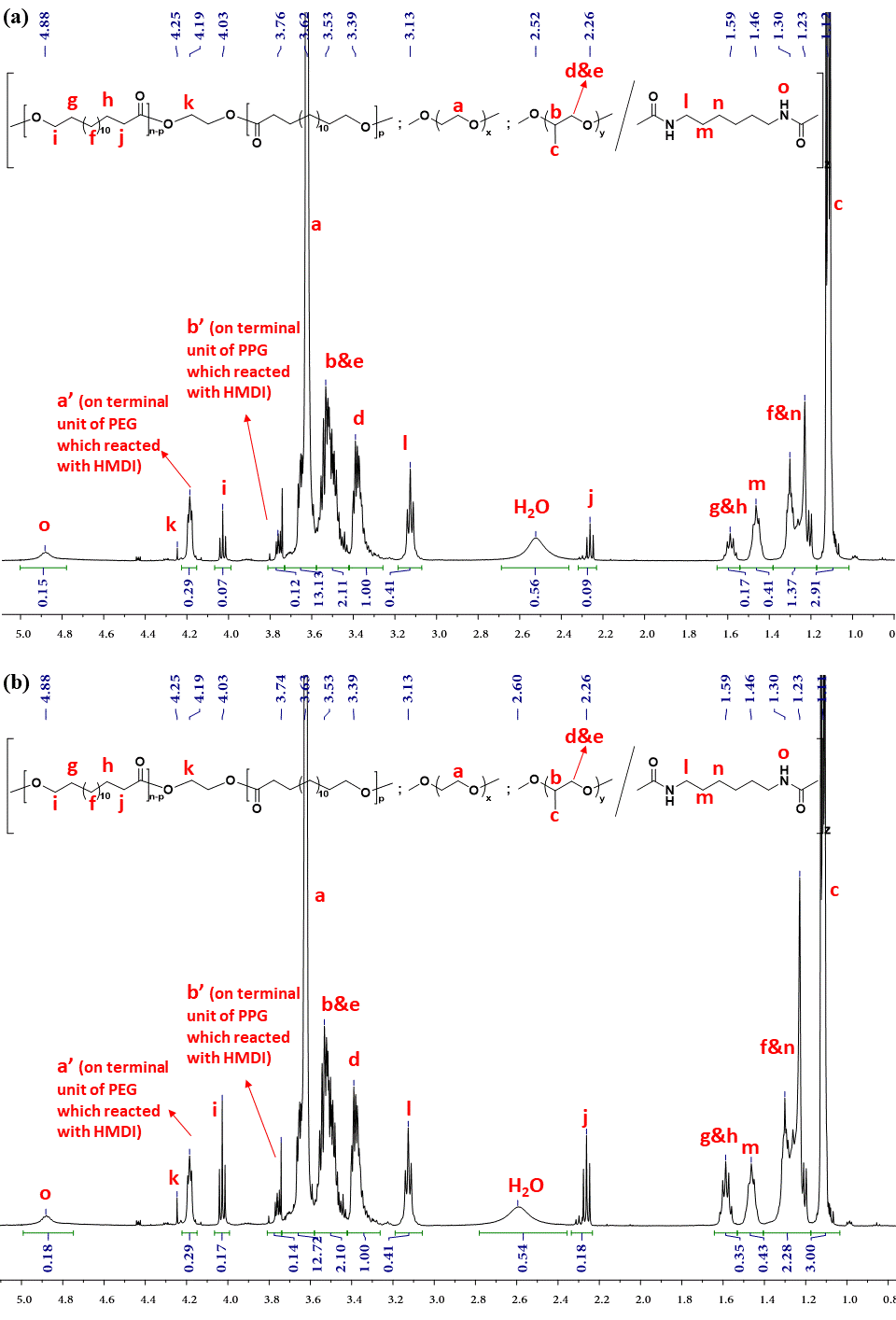


**Figure S1.** 500 MHz ^1^H NMR spectrum of (a) 5PDEP and (b) 8PDEP in CDCl_3_.


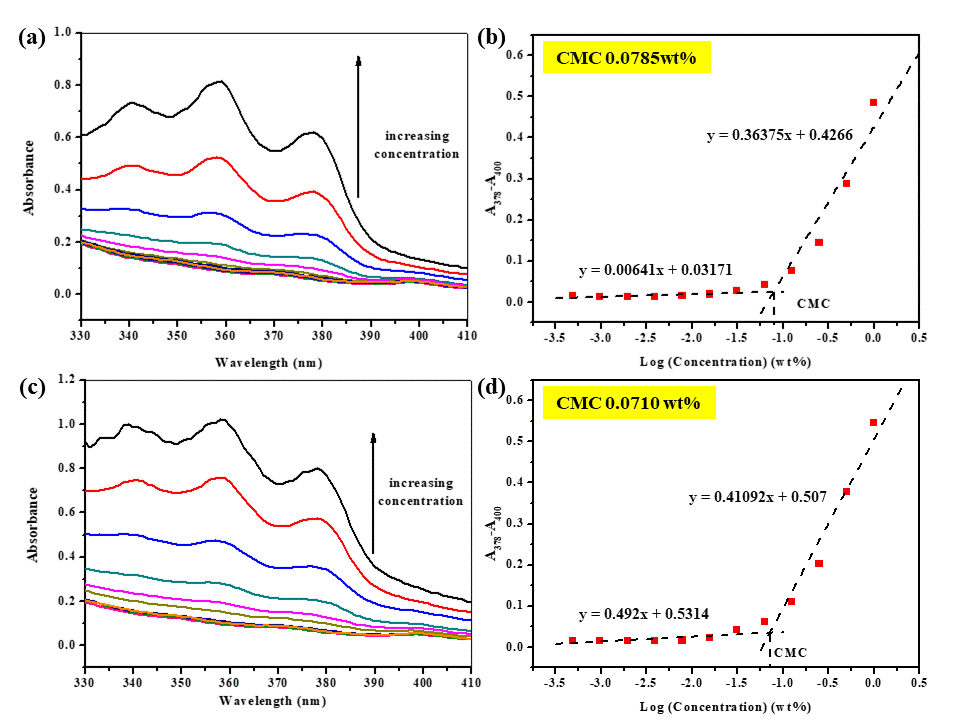


**Figure S2.** (a) UV−vis spectra changes of DPH with increasing concentration ranging from 0.005 to 10 mg·mL^-1^ in water at 25 °C and (b) CMC determination for 5PDEP. (c) UV−vis spectra changes of DPH with increasing concentration ranging from 0.005 to 10 mg·mL^-1^ in water at 25 °C and (d) CMC determination for 8PDEP


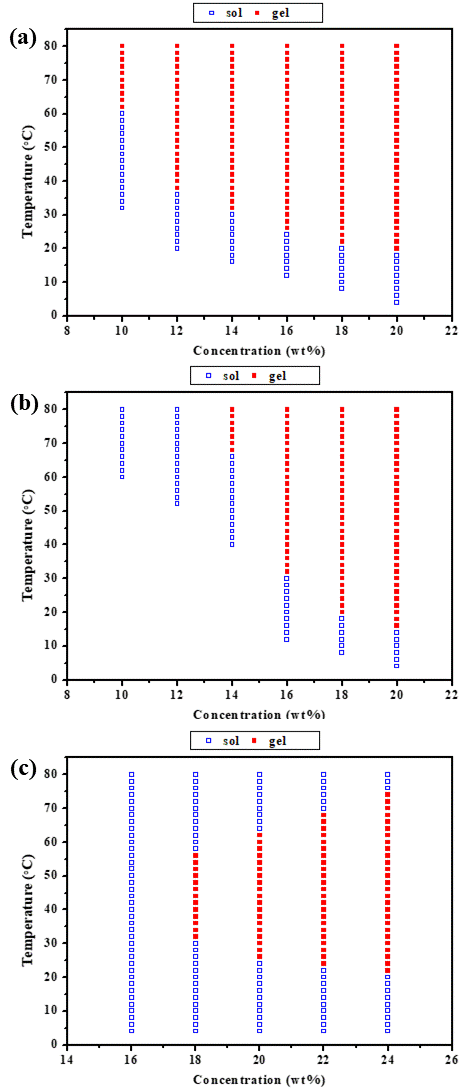


**Figure S3.** Phase diagrams of (a) 5PDEP, (b) 8PDEP and (c) Pluronic^®^ F127.


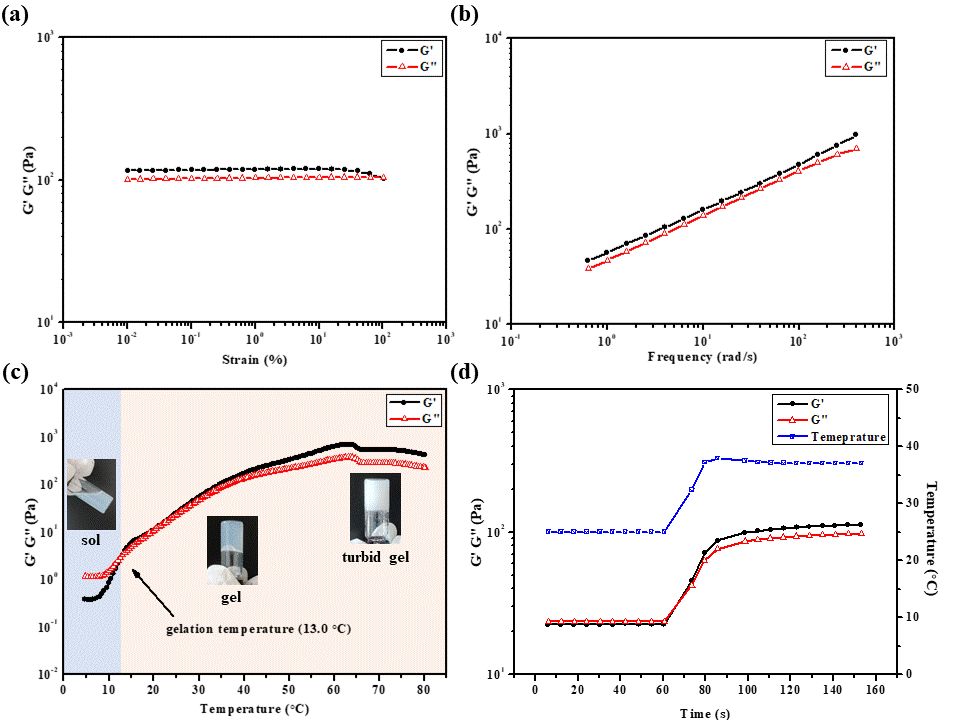


**Figure S4.** Storage modulus (G’) and loss modulus (G”) of (a) amplitude sweep (0.01-100%, 1 Hz, 37 °C), (b) frequency sweep (1%, 0.1-100 Hz, 37 °C), (c) temperature sweep (1%, 1 Hz, 4-80 °C) and (d) temperature ramp (1%, 1 Hz, 25 °C to 37 °C) for 5PDEP aqueous solution (15 wt%) obtained from dynamic rheological analysis.


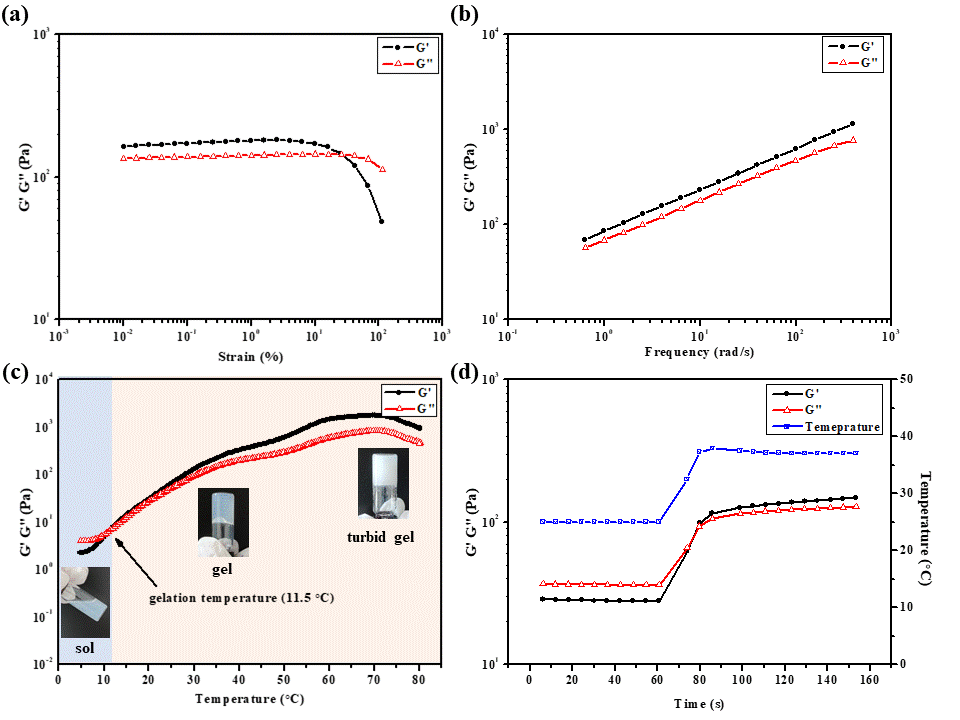


**Figure S5.** Storage modulus (G’) and loss modulus (G”) of (a) amplitude sweep (0.01-100%, 1 Hz, 37 °C), (b) frequency sweep (1%, 0.1-100 Hz, 37 °C), (c) temperature sweep (1%, 1 Hz, 4-80 °C) and (d) temperature ramp (1%, 1 Hz, 25 °C to 37 °C) for 8PDEP aqueous solution (16 wt%) obtained from dynamic rheological analysis.


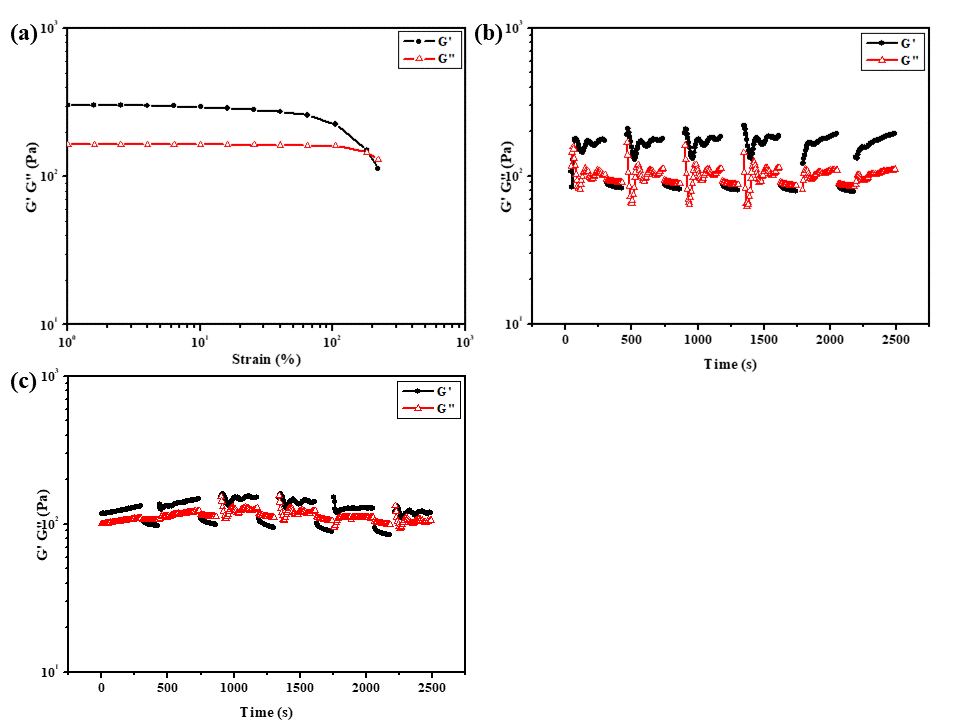


**Figure S6.** (a) Determination of critical break strain for 2PDEP aqueous solution (12 wt%) by amplitude sweep (0.01-200%, 1 Hz, 37 °C) obtained from dynamic rheological analysis. Self-healing cycle amplitude sweep (1 Hz, 37 °C) for (b) 2PDEP aqueous solution (12 wt%) at strain of 0.01% and 200% and (c) 5PDEP aqueous solution (15 wt%) at strain of 0.01% and 95%.
